# Glycemic Efficacy and Safety by Using Insulin Degludec and Aspart Guided by a Clinical Decision Support System in Non-Critically Ill Inpatients with Type 2 Diabetes Mellitus

**DOI:** 10.3390/bios16050289

**Published:** 2026-05-16

**Authors:** Felix Aberer, Daniel A. Hochfellner, Petra M. Baumann, Bernhard Höll, Peter Beck, Thomas R. Pieber, Julia K. Mader

**Affiliations:** 1 Department of Endocrinology and Diabetology, Medical University of Graz, 8036 Graz, Austria; felix.aberer@medunigraz.at (F.A.); petra.baumann@medunigraz.at (P.M.B.); thomas.pieber@medunigraz.at (T.R.P.); julia.mader@medunigraz.at (J.K.M.); 2 Decide Clinical Software GmbH, 8020 Graz, Austria; bernhard.hoell@decide-clinical.com (B.H.); peter.beck@decide-clinical.com (P.B.)

**Keywords:** ultra-long-acting basal insulin, in-hospital glycemic management, type 2 diabetes mellitus, clinical decision support system, continuous glucose monitoring

## Abstract

**Background:** Algorithm-based insulin dosing systems are increasingly used in hospitals and have shown the potential to efficiently and safely enable glycemic control. The goal of this study was to evaluate glycemic control using the ultralong-acting basal insulin degludec (IDeg) in combination with insulin aspart (IAsp) within an algorithm-driven electronic clinical decision support system (cDSS) in inpatients with type 2 diabetes (T2D). **Methods:** In this non-controlled single-arm pilot study, an electronic, algorithm-based cDSS was applied for the management of insulin treatment in an internal general ward. Thirty hospitalized patients with T2D (18 female, age 74.1 ± 10.9 years, HbA1c 72.4 ± 22.3 mmol/mol, BMI 28.6 ± 5.6 kg/m^2^, diabetes duration 13.2 ± 11.6 years, creatinine 1.5 ± 1.2 mg/dL, length of hospital stay 9.1 ± 4.0 days) were included in the study. Capillary blood glucose (BG) was evaluated four times daily using a point-of-care device integrated into the hospital information system. In addition, all participants received a blinded continuous glucose monitoring (CGM; Abbott Freestyle Libre Pro) system. The primary endpoint was defined as the percentage of BG measurements within the target range of 3.9–7.8 mmol/L. **Results:** Overall, 722 BG values and 17,242 CGM data points were available. Of those, 52.2% and 55.0% were in the specified target area (3.9–7.8 mmol/L), respectively. Mean BG prior to study start was 11.9 ± 4.4 mmol/L and improved to 7.5 ± 1.9 mmol/L and 7.4 ± 1.4 mmol/L after 6 and 10 days of treatment. BG < 3.9, <3.0 and <2.2 mmol/L was 1.25%, 0.28% and 0%, respectively. Adherence to the total daily insulin dose suggested by the cDSS was 94.2%, and 99.5% of all basal and 85.3% of all bolus insulin suggestions were accepted by the nurses in charge. Basal-bolus therapy using the cDSS covered 85% of the participants’ total hospital stay. **Conclusions:** Glycemic control using IDeg within an algorithm-driven cDSS could effectively and safely be achieved in the hospital and was highly accepted.

## 1. Introduction

Dysglycemia during the hospital stay contributes to adverse clinical outcomes including prolonged duration of stays, increased risk of complications and mortality. This has been mainly demonstrated by observational data ensuring that higher HbA1c as well as admission and inpatient glucose represent important biomarkers for potential burden [1,2,3,4]. Striving for intensive glycemic control during hospitalization has brought inconclusive and conflicting results in terms of providing certainty that an intensive and tight glycemic control positively impacts different outcome parameters, as risk of hypoglycemia is substantially increased when lower glucose targets are desired to be achieved. As a consequence, current guidelines pragmatically recommend a target glucose range of 7.8–10.0 mmol/L in most of inpatients and lower thresholds (6.1–7.8 mmol/mol) only for those where it can be attained without a risk of relevant hypoglycemia. An insulin regimen employing basal, nutritional and correctional insulin has been identified as the most suitable treatment method for those with relevant hyperglycemia and non-critical illness [5,6].

Algorithm-based, electronic clinical decision support systems (cDSSs) guiding insulin therapy in the hospital have shown to have the potential to improve glycemic control as well as facilitate and standardize workflow issues. Such a cDSS is GlucoTab version 6.0 (decide Clinical Software GmBH, Graz, Austria) which has evidently demonstrated to improve in-hospital glycemia with a low risk of hypoglycemia and persuading user acceptance [7,8,9,10,11]. The previous studies using this cDSS used insulins glargine U100 and U300 [11,12]. Insulin degludec (IDeg) represents an ultralong-acting basal insulin intended to be injected once daily. It is characterized by stable pharmacokinetic and pharmacodynamic properties lasting more than 24 h and is thus associated with low glycemic variability and decreased potential to provoke (nocturnal) hypoglycemia when compared with NPH insulin, insulin detemir or insulin glargine U100 in the outpatient setting [13,14,15,16,17,18,19]. To the best of our knowledge, the efficacy and safety of IDeg when used within a cDSS protocol has not been evaluated yet.

The goal of this feasibility study was to investigate the efficacy and safety of IDeg when guided by a cDSS for insulin therapy in hospitalized, non-critically ill patients with type 2 diabetes (T2D) or new-onset hyperglycemia at a general ward of a tertiary care centre. The primary endpoint was defined as the percentage of glucose values in the glycemic target range of 3.9–7.8 mmol/L during treatment.

## 2. Materials and Methods

This was a prospective, single-centre, non-controlled, interventional trial enrolling individuals with T2D or new-onset hyperglycemia requiring insulin therapy being hospitalized for any kind of internal disease. The trial aimed to explore the feasibility, safety, and efficacy of integrating a cDSS with basal insulin (IDeg) and bolus insulin (IAsp) for glycemic control in hospitalized patients with T2D. The trial was performed at an internal general ward (Endocrinology and Diabetology) in a tertiary care centre (Medical University of Graz). Ethical approval was obtained from the Ethics Committee of the Medical University of Graz (Number: 30-491 ex 17/18; Approval date: 17 October 2018) and the study was registered at the European Union Drug Regulating Authorities Clinical Trials (EudraCT number: 2018-002646-36). Individuals with type 2 diabetes meeting the ADA criteria for insulin therapy [5] in the management of inpatient hyperglycemia were approached for participation and enrolled during their hospitalization in an internal medicine ward. After detailed information on the study procedures, participants signed an informed consent prior to any study investigation. The study was performed in full accordance with the requirements stated in the “Declaration of Helsinki”. All participating study workers, such as physicians, nurses and investigators were instructed in terms of adhering to the study protocol, study-specific tasks, functionality of the cDSS system before being involved in study activities.

### 2.1. Study Procedure

The main objective of the trial was to evaluate the efficacy in achieving an in-hospital glycemic target of 3.9–7.8 mmol/L guided by a cDSS-driven basal-bolus insulin therapy algorithm comprising the use of IDeg as basal insulin in hospitalized patients with T2D at a general internal medicine ward. Secondary objectives were the investigation of the percentage of glucose values within different target ranges, the number and severity of hypoglycemic events and the personnel’s acceptance as well as adherence to dosing recommendations given by the cDSS. Hospitalized patients admitted to the ward of Endocrinology and Diabetology were eligible to participate in the trial. Main inclusion criteria were: age ≥ 18 years, previously known T2D or newly diagnosed hyperglycemia requiring subcutaneous insulin therapy (patients with suspicious features of type 1 diabetes, including ketoacidosis were not considered for the study). Most important exclusion criteria comprised the presence of diabetes mellitus type 1 (T1D), pregnancy or childbearing potential, parenteral nutrition, known allergy to insulin degludec, any mental condition rendering the patient incapable of giving consent, continuous insulin infusion therapy or involvement in another study. Oral insulin secretagogues or pioglitazone were discontinued during cDSS treatment according to the intended use of the device; other oral antihyperglycemic agents (OHAs) and GLP-1-receptor agonists were proceeded, discontinued or newly introduced under the discretion of the practitioner in charge. Initiation and duration of cDSS-based insulin treatment within the study were decided upon the treating physician’s decision with a maximum study duration of three weeks. The discharge therapy including diabetes therapy was established according to the physician in charge.

By utilizing a point-of-care testing (POCT) device (Roche AccuChek^®^ Inform II System, Roche Diagnostics, Rotkreuz, Switzerland), capillary prick measurements were taken at least prior to main meals and at bedtime by the nursing staff in charge. After automatic transmission of these measurements to the laboratory information system, they were forwarded and imported into the cDSS system.

In addition to regular POCT measurements all patients were equipped with a blinded (professional) continuous glucose monitoring system (Freestyle Libre Pro, Abbott Diabetes Care, Alameda, CA, USA) which is factory calibrated, intended to be used for 14 days and providing retrospective professional glucose data visualization and interpretation. The data download was performed after the study termination indicating that glucose values gained from the glucose monitoring system had no influence on therapeutic suggestions.

### 2.2. cDSS-Based Glycemic Management

The cDSS GlucoTab (decide Clinical software GmbH, Graz, Austria) represents a digital workflow system intended for glycemic management in the hospital incorporated into the hospital data system. This cDSS provides a basal-bolus insulin algorithm which aims to achieve fasting and pre-meal BG values of 5.6–7.8 mmol/L [20]. The details of the cDSS and its algorithm, which was not specifically adjusted for this trial, are described in a previously published study of our research group [9]. Briefly summarized, an initial total daily insulin dose is calculated taking patient age, kidney function and body weight into account once a patient is registered in the system. Basal and bolus insulin is recommended in a proportion of 50:50%; bolus insulin is given in advance to scheduled meals. So far, all rapid acting insulin analogues and basal insulins glargine U100 and U300 have been tested and approved for the use within the cDSS algorithm. This study used IDeg as basal insulin in the hospital setting within the algorithm- driven cDSS system. Insulin aspart (IAsp) was used as rapid acting insulin (both insulins: Novo Nordisk, Bagsvaerd, Denmark). Total daily insulin dose suggestions provided by the systems are based on the capillary blood glucose (BG) values of the last 24 h and need to be confirmed once per day by the physician in charge. After every BG measurement (morning, noon, evening, bedtime) which was entered to the system the nursing receives an insulin dose suggestion considering whether a standardized meal is scheduled or not. The medical and nursing staff were authorized to overrule the proposed insulin dose and perform additional glucose measurements any time, if deemed necessary.

### 2.3. Statistical Analysis

Only full 24 h treatment days were taken into account for analysis of efficacy parameters. At least 70% of the CGM measurements had to be available per day to be eligible for analysis of the CGM data of the depicted day. In addition, a minimum of 2 days of sufficient CGM data, had to be available per participant to consider the individual person for data analysis. CGM data was analyzed based on the suggestions for standardizing the analysis and illustration of CGM data [21]. A formal sample size calculation was conducted to test the study hypothesis by using a one-tailed t-test weighted by the total number of glucose measurements per participant, with a power of 80% and a 5% level of significance in order to test whether the mean percentage of capillary BG measurements in the chosen target range of 3.9–7.8 mmol/L (primary outcome) was exceeding the recent best-practice trial with the criterion threshold of 42% [22]. To fulfil the criterion of statistical sufficiency, 27 participants were determined to be enrolled in the study. In order to correct for potential study drop outs, the number of participants in the trial was enhanced to 30. Before data analysis, all metric variables were proofed for normality by means of a Shapiro–Wilk test. The level of significance was defined with 5% for all the tests. Statistical analysis was done using R version 3.1.2 programme.

## 3. Results

Thirty patients were included in the study. Baseline characteristics are given in Table 1. Time to inclusion was 0.6 (0.1–1.2) days. Duration of treatment with the cDSS-based algorithm using IDeg and insulin aspart was 6.2 ± 3.7 days, indicating that the cDSS treatment covered 80.9 ± 19.9% of the patients’ total hospital stay.

### 3.1. Glycemic Control Achieved by cDSS

**Figure 1:**
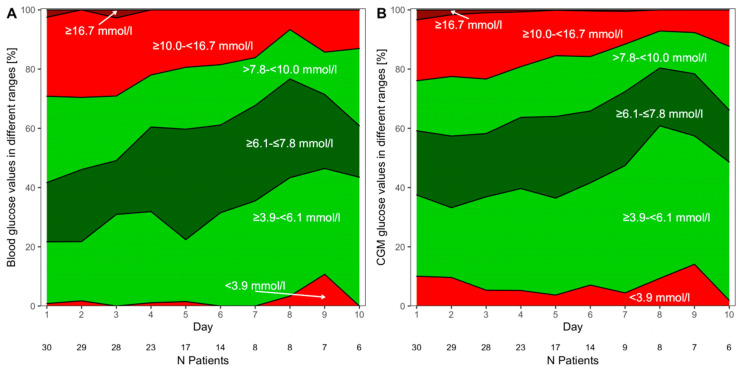
Mean percentage of capillary (**A**) and continuous glucose monitoring (**B**) glucose values in different glycemic ranges during cDSS treatment using IDeg and IAsp. The numbers below the *x*-axis indicate the patient count.

When cDSS was used in combination with IDeg and IAsp within the study procedure, the percentage of BG values (n = 722) being in the predefined target range (3.9–7.8 mmol/L) was 52.2 ± 4.1% and 55.0 ± 3.2% when assessed retrospectively by CGM (n = 17,242). Considering a target range of 3.9–10.0 mmol/L, 74.8% of all capillary BG and 74.2% of CGM measurements were in this range. Mean daily capillary BG during the entire treatment period was 8.4 ± 1.5 mmol/L. Figure 1A,B illustrate the evolution of mean BG and CGM glucose over the course of treatment.

### 3.2. Mean Blood Glucose

**Figure 2:**
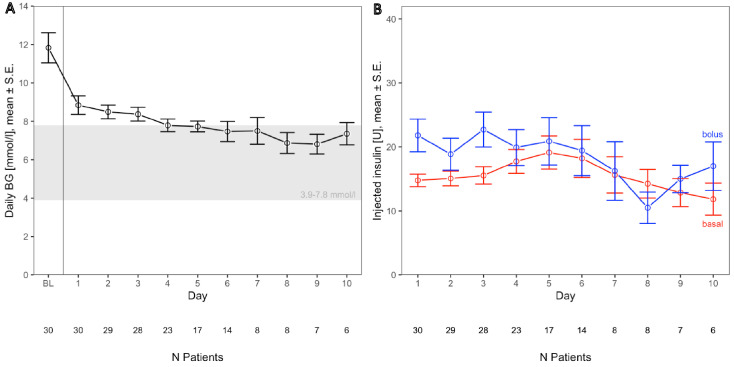
Mean daily blood glucose (± SD) as a function of treatment day (**A**). Day BL (baseline) relates to mean glycemia prior to treatment with the cDSS. Changes of injected basal (red) and bolus (blue) insulin administered by suggestions of the cDSS (**B**) during the according days on the *x*-axis. The numbers below the *x*-axis indicate the remaining count of patients in the study.

Mean BG at baseline prior to study enrolment was 11.9 ± 4.4 mmol/L. During the study, this was reduced to mean BG of (8.4 ± 1.5 mmol/L) in response to cDSS treatment. Figure 2A shows day-by-day changes in mean BG (note that the number of patients decreases severely over time). Distribution of basal and bolus insulin over the period of treatment is shown in Figure 2B.

### 3.3. Glycemic Variability

**Figure 3:**
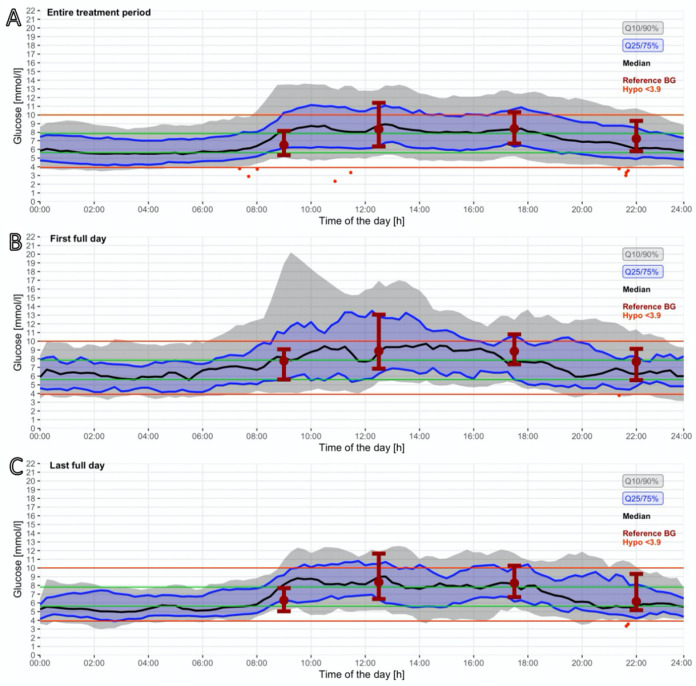
Evolution of CGM data over the day. (**A**) Total treatment period. (**B**) First full treatment day. (**C**) Last full treatment day. The red lines indicate the target of 3.9–10.00 mmol/L, the green lines indicate the target. The green lines indicate the stricter target range of 6.1–7.8 mmol/L.

GV was also reduced from first full treatment day (33.0%) to last (31.8%) (Figure 3).

### 3.4. Safety of the cDSS

In total, nine hypoglycemic episodes <3.9 mmol/L in seven participants determined by capillary BG measurements occurred, which corresponds to a percentage of 1.25% of all available BG values (n = 722). Regarding CGM data, 7.1% of all values were <3.9 mmol/L during the overall trial period. Comparing the first vs. the last complete treatment day, percentage of hypoglycemic values < 3.9 mmol/L recorded by CGM decreased over time, but low values occurred in more patients (10.0% in 19 patients vs. 8.8% in 21 patients). On the first complete study day, 0.8% of BG measurements in one patient were <3.9 mmol/L, compared to 1.7% in two patients on the last full study day. None of the hypoglycemic events led to the necessity of third-party help or resulted in a serious adverse event. Of note, not a single BG value was <2.2 mmol/L.

### 3.5. Adherence to the cDSS Advice

Adherence by physicians to insulin dose suggestions was 94.2%; the nursing staff accepted 99.5% of basal and 85.0% of bolus insulin dose suggestions. If adjustments were done by the medical staff, the changes were small, typically reductions and mostly concerned bolus insulin: median (Q1–Q3) bolus correction −2.0 (−3.5; −1.0) IU (n = 67 corrections). Five adjustments to basal insulin suggestions were made; four of them occurred on the first day of the study, thus before the first complete study day. The nursing staff carried out 100% of basal and 100% of bolus injections, and 98.2% of all mandatory BG measurements (n = 722/735) were conducted, while 28 additional BG measures were taken beyond the four scheduled measurements per day.

## 4. Discussion

This feasibility study investigated the efficacy and safety of using IDeg as a component of basal-bolus insulin therapy guided by a cDSS in non-critically ill hospitalized patients with either T2D or new-onset hyperglycemia requiring subcutaneous insulin therapy.

Of 722 available BG values, 52% were in the selected target range of 3.9–7.8 mmol/L. When assessed by CGM, of more than 17,000 data points, 55% were in this target. This chosen target range was derived from a best-practice study in which a basal-bolus insulin titration protocol was compared against a basal-plus regimen in hospitalized patients with T2D [22]. In this study, Umpierrez et al. reported a percentage of about 42% of all BG values being in this pre-specified target range, indicating superior performance when our data is considered. A study in a similar setting of hospitalized patients with T2D on general and surgical wards comparing insulin IDeg with insulin glargine U100 revealed a percentage of values in range of 3.9–10 mmol/L of 54.5 ± 29.0% for IDeg and 55.3 ± 28.0% for glargine U100 when the insulin dosing was conducted under the discretion of the primary care team [23]. In our study, cDSS-guided insulin therapy with IDeg achieved 74.5% of all values in this range (3.9–10 mmol/L). The percentage of glucose values in the hypoglycemic range was not reported in this study. However, in our study, only 1.25% of all capillary glucose values were classified as hypoglycemic (none of them classified as severe hypoglycemia) allowing the conclusion that cDSS-guided therapy applying IDeg can be considered effective and safe. We are aware that the target range in the hospital according to the guidelines of the American Diabetes Association, is chosen higher in the majority of people (7.8–10 mmol/mol) and a lower target BG goal (6.1–7.8 mmol/mol) should be only recommended in people as long as they achieve it without significant hypoglycemia. These very cautious therapy goals are mainly based on lacking evidence supporting a clear benefit of intensive glycemic control attributing to beneficial clinical outcomes. Hypoglycemia, which inevitably occurs more frequently when lower glycemic targets are aimed to be achieved, has been discussed to be the main driver impacting adverse outcome as it has been shown in ICU and non-ICU settings as well as in surgical and medical inpatients [4,24,25,26,27]. Indeed, inpatient hypoglycemia remains an important issue which cannot be ignored: observational data provided from the UK National Diabetes Audit 2017 reported about 18% of patients with diabetes experiencing at least one hypoglycemic event during the hospital stay, whereas of those, 7% were classified as severe hypoglycemic episodes requiring third-party assistance [28]. The incidence of hypoglycemic events in clinical trials applying subcutaneous insulin was estimated with 1 to 32% depending on hypoglycemia definition and employed glucose-lowering therapy. Guiding insulin therapy with an algorithm-based electronic decision support system for inpatient glycemic management (GlucoTab) has previously demonstrated a low rate of hypoglycemia: When using insulin glargine U100 in 99 hospitalized non-critically ill patients with T2D, percentage of hypoglycemic BG values was 1.5% [9]; when insulin glargine U300 was used, hypoglycemia < 3.9 mmol/L occurred in about 1.0% [11] which is comparable to the exhibited hypoglycemic percentage in this study investigating the other novel ultralong-acting IDeg (1.25%). Hypoglycemia was detected in a higher proportion of the available glucose values (7.1%) when assessed by CGM, for which data was downloaded in retrospect. This has been shown previously by Gomez et al. who reported about a 4 times higher number of determined hypoglycemic events when CGM was compared against POCT measurements in a study which employed basal-bolus insulin therapy in hospitalized patients with T2D [29]. Despite the authors in the paper concluding that these CGM values were clinically valid, a percentage of 91.9% outside the zones A and B in the Clarke Error Grid indicate a non-ignorable remaining 8.1% of reference measurements that need to be taken with caution regarding their accuracy. The limitations of using CGM as a comparator to POCT measurements have been summarized in an article by Schrangl et al. who report on methodical concerns when statements about the quality of glycemic control are based on CGM values [30]. The CGM system used for this study, Abbott Freestyle Libre Pro, has been recently evaluated in an in-hospital setting in insulin-treated patients with T2D. The CGM tended to underestimate glycemia during all glucose values (mean glycemia assessed by POCT 10.49 mmol/L and by CGM 9.78 mmol/L), revealed a higher proportion of time below 3.9 mmol/L (1.1% assessed by POCT vs. 4.5% assessed by CGM) and exhibited a mean absolute relative difference (MARD) of almost 28% in the hypoglycemic glucose range which can be regarded as quite inaccurate [31]. In our study, not a single hypoglycemic event was classified as severe (<2.2 mmol/L) and/or required third-party help.

The low rate of hypoglycemic glucose values while achieving sufficient glycemic control might be attributed to the use of IDeg as basal-insulin in addition to nutritional or correctional insulin when glucose control was established under the guidance of a clinical decision support system.

Observational trials have indicated that the use of IDeg in the hospital enables sufficient glycemic control, characterized by increased percentage of glucose values in target during the course of the hospital stay, more balanced glycemic variability and being associated with a very low risk of hypoglycemia when suggested by a standardized insulin titration protocol [32,33]. In our prospective study we were able to replicate these data and could provide more insight into glucose profiles by employing continuous glucose monitoring during the study.

The molecular structure of IDeg allows it to reversibly bind to albumin, potentiating the ability to buffer absorption rate changes. The primary mechanism of protraction consists of monomers which are released at a slow and steady rate after the injection and despite being the only basal insulin which bears a half-life > 25 h; the dosing interval can be relatively variable due to the fact that the pharmacokinetic properties diminish accumulation effects [34]. Especially in inpatients, over-punctual dosing of basal insulin can be challenging due to the fact that nurses’ workloads are often irregular and the timing of insulin injections are frequently interrupted by emergencies. Thus, IDeg use in hospitals might offer more flexibility in terms of adhering to the timing of basal insulin administration.

The user acceptance and adherence to suggestions deriving from a medical product are crucial in everyday daily routine. A dose adherence to 99.5% of all basal-insulin and 85.3% of all bolus-insulin suggestions in this study underlines user confidence by working with the system. Similar results related to usability have been shown in previous studies in which the presently investigated cDSS was used [8,9,11]. Of note, this study was conducted in an endocrinology ward where diabetes specialists are entrusted with the task of managing glycemic control. Potentially, glucose control is taken more seriously in such settings and user acceptance might be diminished when the system is employed in a non-medical ward compared to surgical departments where diabetes is regarded as an inconvenient comorbidity that does not receive intensive medical dedication. However, user acceptance towards the cDSS was also high in a surgical ward as shown in a previous study, and interestingly, time in target range was highest in this surgical ward when compared to three of the medical wards of the centre [9].

The majority of participating patients (n = 26) were not immediately treated according to the study intervention comprising IDeg within the algorithm-based treatment provided by the cDSS (median time until study inclusion was 0.55 ± 1.96 days) at admission but supplied with glucose-lowering therapy as recommended by the physicians in charge. Our data indicate a continuous improvement of time in target during the course of the hospital stay. While median pre-enrollment glycemia was 12.3 ± 4.4 mmol/L, glucose improved to 8.8 ± 2.6 mmol/L after the first study day and to 7.2 ± 1.9 mmol/L at day 6 which almost half of the patients (n = 14) experienced. It has been shown in the past that physician-based recommendations for continuous titration of glycemic therapy are commonly hesitant and reluctant, contributing to a failure to control hyperglycemia in hospitalized patients [35]. The algorithm-based therapy provided by the cDSS allows a consequent adjustment of insulin therapy leading to subsequent improvement of glycemic control. Interestingly, while we previously observed a steady increase in doses of insulin glargine U300 over the course of the study (total daily dose [TDD] at first full study day 35.6 (24.9–46.3) vs. 103.6 (36.8–170.3) at last full study day), in this trial using insulin IDeg, the doses remained almost unchanged throughout the days of observation (total daily dose at first full study day was 32.5 (25.0–43.8) vs. 28.5 (22.5–50.3) U at last full study day). Of note, these TDD values were overall much lower when compared to U300. This finding might be patient-selection based (Baseline HbA1c was higher in the U300 study [78 mmol/mol in U300 vs. 72 mmol/mol in IDeg] and a higher proportion of patients in the U300 study had insulin therapy already [20 in U300 and 17 in IDeg] at baseline), unlikely to be associated with the kinetics of IDeg but needs further attention in future. Higher day-to-day glucose variability (GV) contributes to an increased risk of severe hypoglycemia, major cardiovascular events and all-cause mortality, as seen in the DEVOTE study where capillary fasting-glucose measurements were considered to assess GV [36]. Since the availability of CGM systems which nicely illustrate glycemic fluctuations, GV moved even more into the spotlight as potential predictor of diabetes-associated complications. Higher CGM-measured in-hospital GV has been shown to negatively impact latter cardiovascular and morbidity and mortality [37,38]. IDeg has previously been shown to contribute to less GV when compared to insulin glargine U100 and insulin detemir in people with T2D [39] and had a non-inferior GV when compared to insulin glargine U300 in people with T1D [40] and T2D [41]. In this depicted study GV was reduced from 33.0% to 31.8% when first and last full treatment day were compared.

Some limitations of this study have to be acknowledged. First, the study was a single-arm study that does not provide an alternative intervention-arm with which to compare. However, given the fact that performance was similarly effective as it was seen using insulin glargine U100 and U300 [9,11], it can be assumed that the algorithm does not have to be adapted in response to using IDeg within the cDSS. Secondly, the sample size (n = 30) was rather small, with a reduction in sample size over time; nevertheless we consider this number of participants as sufficient in providing a thorough insight into 24 h profiles of CGM data as shown in other studies applying a cDSS for inpatient diabetes management [8,9,11,42]. Next, the study was conducted at an endocrinology ward, which employs diabetes specialists, and the nursing staff is familiar with the use of the cDSS used. In summary, insulin therapy using the cDSS with IDeg and IAsp was effective in achieving glucose targets with a low risk of hypoglycemia and a well accepted usability.

## 5. Conclusions

cDSS-guided basal-bolus insulin therapy using IDeg and IAsp was demonstrated to be effective and safe in non-critically ill hospitalized patients with T2D or new-onset hyperglycemia in this pilot study. The approach achieved a high proportion of glucose values within target, with minimal hypoglycemia, and was well accepted by clinical staff. These preliminary findings support the feasibility of integrating IDeg into algorithm-based inpatient glycemic management. Future studies should explore larger, multi-centre populations and evaluate clinical outcomes beyond glycemic control.

## Figures and Tables

**Table 1:** Baseline characteristics; BMI = Body Mass Index, OHAs = oral hypoglycemic agents.

Participants (n)	30
Sex f/m (n (%))	18 (60)/12 (40)
Age (years)	74.1 ± 10.9
BMI (kg/m^2^)	28.6 ± 5.6
Weight (kg)	80.3 ± 19.1
Race: Caucasian/African/Asian	30/0/0
Serum creatinine (mg/dL)	1.5 ± 1.2
HbA1c (mmol/mol)	72.4 ± 22.3
Duration of diabetes (years)	13.2 ± 11.6
Admission reason: acute/scheduled (n (%))	29 (96.7)/1 (3.3)
**Admission therapy (n (%))**	
Newly diagnosed or diet only	2 (6.7)
OHA only	11 (36.7)
Insulin only	4 (13.3)
Insulin and OHA	13 (43.3)

## Data Availability

The data supporting the findings of this study are available from the corresponding author upon reasonable request. Due to privacy and ethical considerations, the data are not publicly available; however, they can be shared with qualified researchers for scientific purposes if required.

## Abbreviations

The following abbreviations are used in this manuscript:

CGM	Continuous Glucose Monitor
cDSS	Clinical Decision Support System
T2D	Type 2 Diabetes
IAsp	Insulin Aspart
IDeg	Insulin Degludec
BG	Blood Glucose
POCT	Point-of-care Testing
GLP1	Glucagon like peptide 1
GV	Glycemic variability
